# Mineral composition, the profile of phenolic compounds, organic acids, sugar and *in vitro* antioxidant capacity, and antimicrobial activity of organic extracts of *Juniperus drupacea* fruits

**DOI:** 10.1002/fsn3.3586

**Published:** 2023-07-30

**Authors:** Hatice Feyza Akbulut, Mehmet Akbulut

**Affiliations:** ^1^ Department of Medicinal and Aromatic Plants, Cumra Vocational School Selçuk University Konya Turkey; ^2^ Department of Food Engineering, Agriculture Faculty Selcuk University Konya Turkey

**Keywords:** antimicrobial activity, antioxidant capacity, *J. drupacea*, minerals, organic acids, phenolic compounds

## Abstract

*Juniperus drupacea* fruit is widely used in traditional and complementary medicine in Turkey for the treatment of different diseases in various forms such as molasses and tar. This study was carried out to evaluate the phenolic compounds, organic acid, sugar, and macro‐ and micromineral distributions of methanol and water extracts of *J. drupace* fruit, as well as their antioxidant and antimicrobial potential. For this purpose, total phenolic content by spectrophotometer, phenolics, organic acids, and sugars distributions by HPLC in extracts of *J. drupacea* fruits, and macro‐ and micromineral element content by ICP‐AES in fruit were determined. 2,2‐diphenyl‐l‐picrylhydrazyl assay (DPPH assay) was used to evaluate in vitro antioxidant activity in extracts. The antimicrobial potential of *J. drupacea* fruit methanol extract against some gram‐positive and gram‐negative pathogenic bacteria was evaluated using disk diffusion and minimum inhibitory concentration (MIC) methods. The potassium macroelement and the iron microelement were found at high content in *J. drupacea* fruit. The total phenolic content in the methanol extracts was higher than the water extracts. Among the individual phenolic compounds, catechin, a flavonoid that was the highest in both extractions, was determined as 300.49 μg/g in methanol extract and 314.88 μg/g in water extract. DPPH scavenging activity was higher in methanol extracts. While the methanol extract of *J. drupacea* had no‐inhibitory effect on the gram‐negative bacteria tested, it exhibited a strong inhibition on the gram‐positive bacteria *Listeria innocua*, *Listeria monocytogenes*, *Staphylococcus carnosus*, and *Enterococcus faecalis*.

## Graphical abstract

This study was carried out to evaluate the phenolic components, organic acid, sugar, and macro‐ and micromineral distributions of methanol, and water extracts of *Juniperus drupacea* fruit, as well as their antioxidant and antimicrobial potential. Among the individual phenolic compounds, catechin, a flavonoid that was the highest in both extractions, was determined as 300.49 μg/g in methanol extract and 314.88 μg/g in water extract. DPPH scavenging activity was higher in methanol extracts. While the methanol extract of *J. drupacea* had no‐inhibitory effect on the gram‐negative bacteria tested, it exhibited a strong inhibition on the gram‐positive bacteria *Listeria innocua*, Listeria.

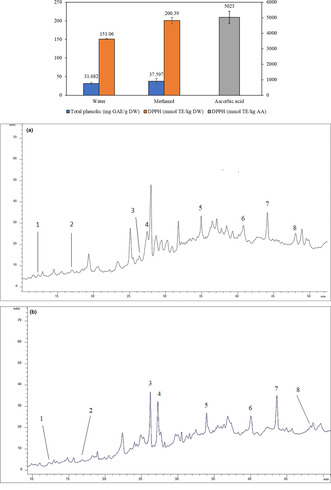

## 1 INTRODUCTION

*Juniperus drupacea* is one of the juniper species and grows wild in countries such as Syria, Lebanon, and Greece in the Mediterranean region, especially in Turkey. It is widespread in the mountainous areas of the Eastern and Central Mediterranean region of Turkey, in areas with an altitude between 600 and 1750 m.

Maturated fruits (cones) of the *J. drupacea* tree have a red‐brown color and a bluish‐waxy layer on them. *J. drupacea* fruits have a very important place in Turkey. The local people have been producing molasses (pekmez) from the fleshy parts of the fruit for centuries and have used this molasses and the tar obtained from the cones for treatment in traditional medicine (Gültekin, 2014).

*J. drupacea* fruits are widely used for therapeutic purposes in traditional medicine in Türkiye (Akkol et al., 2009). For example, the fruits of *J. drupacea* have been used to treat helminth infections and abdominal pain (Honda et al., 1996), against hemorrhoids (Baser et al., 1986), and the decoction of fresh shoots for the treatment of urinary inflammation, abdominal pain, and gout, and tar of this species has been used against diarrhea (Yeşilada et al., 1995). At the same time, *J. drupacea* fruit methanol and water extracts have been reported that they had positive effects on COPD markers (Akbulut, 2022).

*J. drupacea* tar is prepared by burning its stem and has been used topically in eczema, alopecia, and animal wounds, and internally for the treatment of cough, cold, urinary inflammation, and diarrhea (Baytop, 1999).

Phytochemicals are bioactive compounds that are formed as a result of the secondary metabolic activities of plants and have very beneficial effects in terms of human health. The most known phytochemical compounds are especially phenolic compounds, tannins, saponins, carotenoids, coumarins, tocopherols, terpenes, isothiocyanates, sulfites, sulforaphanes, terpenoids, alkaloids, flavonoids, phytosterols, phytoestrogens, and indoles (Fidan & Dündar, 2007).

Phenolic compounds consist of two groups: flavonoids and phenolic acids. The widest group of naturally occurring polyphenols is flavonoids, including flavones, flavonols, flavanones, flavanols, isoflavonoides, and anthocyanidins (Salehi et al., 2019). These flavonoid group compounds act as free radical scavengers and antioxidants with antimutagenic, anti‐inflammatory, antiviral, anticarcinogenic, and antimicrobial effects (Coklar & Akbulut, 2017, 2019, 2021; Shankar et al., 2017; Senhaji et al., 2022).

There are a few studies on *J. drupacea* in the literature, and the its fruits used in these studies were obtained from the forests of Antalya and Kahramanmaraş provinces of Turkey at an altitude of 1000 m. In our study, *J. drupacea* fruits collected from the forests at an altitude of 1400 m of Mersin Çamlıyayla region of Turkey were examined for the first time. In addition, there are no studies on the organic acid and sugar distribution of *J. drupacea*. At the same time, it is seen that the antimicrobial effects of *J. drupacea* extracts have not been tested on *Staphylococcus carnosus*, *Bacillus cereus*, *Listeria monocytogenes*, *Escherichia coli*, *Listeria innocua*, *Klebsiella pneumoniae*, and *Enterococcus faecalis* pathogenic bacteria in the relevant literature, except for *S. aureus*. In this respect, this study was carried out to fill the gaps left in the literature about *J. drupacea* fruit. In this study, it was aimed to determine the phenolic, organic acid, and sugar distributions of methanol and water extracts of *J. drupace* fruit and to reveal the in vitro antioxidant capacity and antimicrobial activity potential. In addition to all these, macro‐ and micromineral elements of *J. drupace* fruit were determined, and it was ensured that it constituted a reference for further studies on health.

## 2 MATERIALS AND METHODS

### 2.1 Chemical and reagents

Gallic acid, protocatechuic acid, *p*‐hydroxybenzoic acid, catechin, epicatechin, *p*‐coumaric acid, epicatechin gallate, procyanidin A2, sucrose (D‐(+) saccharose), fructose (D‐fructose), and glucose (D‐glucose) were purchased from Extrasynthese (Genay, France). Citric, tartaric, malic, lactic, formic, and propionic acid standard reagent were obtained from Bio‐Rad (Istanbul, Turkiye). Acetonitrile (HPLC grade) from Honeywell, ethyl acetate, methanol, and Folin–Ciocalteu's reagent from Sigma‐Aldrich (Steinheim, Germany), acetic acid, sodium carbonate, nitric acid, ascorbic acid, and 2,2‐diphenyl‐1‐picrylhydrazyl (DPPH) from Merck (Darmstadt, Germany) were acquired. Ampicillin, vancomycin, penicillin G, netilmicin, ciprofloxacin, and gentamicin antimicrobial susceptibility test disks were purchased from Bioanalyse (Ankara, Turkiye). Ultrapure water used in extraction and analyses was obtained through Millipore Direct‐Q 3 UV ultrapure water system (Millipore, USA).

### 2.2 Material

#### 2.2.1 Plant material

*J. drupacea* Labill. species (Cupressaceae) were collected from forest of Sebil in the area of Mersin Çamlıyayla province in Turkey in July. The plant materials were identified by Prof. Dr. Osman TUGAY from the Department of Pharmaceutical Botany, Faculty of Pharmacy, Selçuk University (Konya, Turkey) and given a Herbarium number (KNYA Herb. No: 30.115).

#### 2.2.2 Extraction of *J. drupacea* fruits

*J. drupacea* Labill. fruits, which has reached the appropriate harvest maturity, were collected from the forest at an altitude of 1400 m in the Çamlıyayla district of Mersin in November 2020 and transferred to the laboratory. The fruits were cleaned of all kinds of impurities. After the fruits were broken, they were ground by a suitable hammer mill (Arzum brand AR1034, Turkiye). The powdered fruits were used in extraction. Ground fruits (15 g) were extracted separately with methanol (150 mL) and water (150 mL) using a Soxlet unit (Electro‐mag MX 425, Turkiye). The methanol and water extracts were collected separately, and the solvents were removed in the rotary evaporator under the vacuum at 40°C (SCILOGEX RE‐100 pro). Afterward, the extracts were frozen at −80°C and lyophilized (Labogene ScanVac Coolsafe110‐4, Lynge, Denmark). Prepared samples were stored at −18°C until analyses. Methanol and water extraction yields of *J. drupacea* were %27.96 ± 1.25 and %25.35 ± 1.25, respectively (*p*‐value .012; *p* < .05).

### 2.3 Determination of phenolic content

#### 2.3.1 Total phenolic content

The total phenolic content was determined using the colorimetric Folin–Ciocalteu method, which is based on the formation of colored complexes between phenolic compounds and Folin reagent in alkaline medium (Singleton & Rossi, 1965). After mixing the appropriately diluted *J. drupacea* fruit extracts (0.5 mL) and Folin solution (2.5 mL; 0.2N), Na_2_CO_3_ solution (2 mL; 75 g/L) was added and it waited for 2 h for the reaction to be completed. Then, the samples were read at 765 nm by spectrophotometer. The results were given to mg gallic acid equivalent (GAE) g^−1^ of DW (dry weight).

#### 2.3.2 Phenolic profile analyses

To determine phenolic profiles of *J. drupacea* fruit extracts, purification was carried out using a C18 SPE cartridge to eliminate sugars and organic acids from the extracts and obtain phenolics. Two ml of extract was loaded into conditioned C18 SPE cartridges (Agilent, USA) by passing water and methanol. To eliminate non‐phenolic impurities such as organic acids and sugars, water was first passed through the cartridge and then phenolics were separated by passing methanol. Methanol was removed with the aid of a rotary evaporator set at 35°C and then resuspended with 1 mL of methanol and filtered through a 0.45‐μm syringe filter (Coklar & Akbulut, 2017). The phenolic compound analysis in *J. drupacea* fruit extracts was performed by an Agilent 1260 Infinity Series HPLC system equipped with DAD detector. Separation was carried out by a reverse phase C18 column (5 μm, 250 × 4.6 mm i.d.). Mobile phase was composed of acetic acid: water (A) and water:acetonitrile:acetic acid (B). Flow rate was 0.75 mL min^−1^, and gradient was as follows: 10%–14% B (5 min), 14%–23% B (11 min), 23%–35% B (5 min), 35–40% B (14 min), 40%–100% B (3 min), 100% B isocratic (3 min), 100%–10% B (3 min), and 10% B isocratic (4 min). The detector was adjusted to 280, 320, and 360 nm to detect phenolics. (Coklar & Akbulut, 2017). In the identification of phenolics, the retention time and UV spectra were evaluated together. External phenolic standards were used for the quantitative determination of each compound. To create calibration curves with standards were used at least five different concentrations, and the validation coefficients (*R* ^2^) of the generated calibration curves ranged from 0.9991 to 1000. Data analysis was carried out with ChemStation software.

### 2.4 Determination of organic acids and sugars profile

Two grams of water and methanol extracts of *J. drupacea* fruit were dissolved in 25 mL of ultrapure water. This dilute extract solution was filtered by a 0.45‐μm pore size syringe filter and then it was sent to HPLC with a volume of 20 μL by autosampler. The detection of organic acids and sugars was performed by an Agilent 1260 Infinity Series HPLC system equipped with DAD detector for organic acids and refractive index (RI) detector for sugars. Separation was carried out by Aminex HPX‐87H column (Bio‐Rad, 300 × 7.8 mm). Mobile phase consisted of 0.005 N sulfuric acid, and the flow rate was 0.6 mL min^−1^. To determine the organic acids, the DAD detector was adjusted to 210 nm. The temperature was held at 50°C (Coklar et al., 2018). The retention times were used to identification of organic acids and sugar. The data were analyzed by ChemStation software.

### 2.5 Macro‐ and micromineral analyses

Approximately 0.5 g dried and ground *J. drupacea* fruit sample was added into a burning cup, and then 15 mL pure HNO_3_ was added. The sample was incinerated in a MARS 5 Microwave Oven at 200°C and dissolved ash was diluted to a certain volume with ultrapure water. Concentrations of macro‐ and micromineral elements were determined by an ICP‐AES (Skujins, 1998).

### 2.6 Antioxidant capacity analyses

The 2,2‐diphenyl‐1‐picrylhydrazyl (DPPH) scavenging capacity of the methanol and water extracts of *J. drupacea* fruit were analyzed with respect to methods defined by Brand‐Williams et al. (1995). According to this method, 0.1 mL aliquots of the fruit extracts were added to 3.9 mL of DPPH (6 × 10^−5^ M) methanolic solution. After 30 min of incubation at room temperature in the dark, the absorbances of the samples were measured with a spectrophotometer set to 515 nm. The results were given as mmol Trolox equivalent/kg DW.

### 2.7 Antimicrobial activity of plant extracts

#### 2.7.1 Tested microorganism

To evaluate antibacterial activities of methanol extracts, eight strains of pathogen bacteria which are *Staphylococcus aureus* ATCC 43300, *S. carnosus* NRLL 14760, *B. cereus* ATCC 14579, *L. monocytogenes* ATCC 13932, *E. coli* ATCC 25922, *L. innocua* ATCC 33090, *K. pneumoniae* ATCC 13883, and *E. faecalis* ATCC 51559, were used. The antibacterial activity study is performed with different and complementary techniques: the disk diffusion method followed by determination of minimum inhibitory concentrations (MIC) (Senhaji et al., 2020).

#### 2.7.2 Disk diffusion method

The antibacterial activity of *J. drupacea* fruit extract was performed according to the method described by Mostafa et al. (2018) with minor modifications and the disk diffusion method was used. Methanol extract of *J. drupacea* fruit was dissolved in distilled water. The methanol extract of *J. drupacea* fruit was dissolved in ultrapure water and then transferred to a 6 mm diameter antimicrobial sensitive blank disk to obtain a final concentration of 20 μL/disk (Bioanalyse, Turkey). Ten milliliters of Muller‐Hilton agar medium was poured into sterile petri dishes, and then, 15 mL of inoculated medium, previously inoculated with bacterial suspension (100 mL of medium/1 mL of 10^7^ CFU), was added to reach 10^5^ CFU/mL. The disks loaded with *J. drupacea* fruit extract concentration of 20 uL/disk were set on the top of Mueller–Hilton agar plates. The distilled water‐treated disks were used as controls. Plates were kept in a refrigerator at +5°C for 2 h for the diffusion of *J. drupacea* extracts. And then they were incubated at 35°C for 48 h. The presence of the inhibition zones formed was measured with the aid of a caliper. The obtained values were recorded, and these were considered as antimicrobial activity. The diameter of the clear zones around each well was measured. Consequently, the inhibition zones between 5 and 10 mm were considered as a strong inhibition (++), and the inhibition zones between 1 and 5 mm (+) were considered as a weak inhibition. The regions without diameter formation were evaluated as no‐inhibition (−) (İspirli et al., 2017).

#### 2.7.3 Determination of minimum inhibitory concentration (MIC)

To determine the minimum inhibitory concentration (MIC) for each test organism, the macrodilution broth method was used. A twofold serial dilution of each *J. drupacea* fruit extract was prepared and followed by dilution in Mueller–Hinton broth to get a decreasing concentration in the range of 50 to 0.39 mg/mL. Each dilution was seeded with 100 μL of the standardized microbial inoculum (1.5 × 10^6^ cfu/mL). The inoculated culture tubes of tested pathogenic bacteria were incubated at 37°C for 24 h. A set of tubes including only broth was kept for the control. And then, incubation tubes were studied for changes in turbidity to be an indicator of growth. The lowest concentration that produced no visible growth was MIC (Dhiman et al., 2021).

### 2.8 Antibiotic susceptibility

Resistance of *L. innocua*, *L. monocytogenes*, *S. auereus*, *S. carnosus*, *B. cereus*, *E. faecalis* against ampicillin (AM 10 μg), vancomycin (VA 30 μg), penicillin G (P 10 UI), and resistance of *E.coli*, *K. pneumoniae* against netilmicin (NET 30 μg), ciprofloxacin (CIP 5 μg), gentamicin (CN 10 μg) was determined using antibiotic disks. *E. faecalis* was inoculated into M17 Broth and the others were inoculated into Brain Hearth Broth and allowed to grow throughout 1 night. One hundred microliters of microorganisms that reached about 0.5 McFarland level were inoculated into petri dishes containing Mueller–Hinton Agar medium. Then, it was spread homogeneously on the petri dish with a Drigalski spatula. Antibiotic disks were placed in the relevant parts of the petri dishes, which were previously divided into sections. After 24 h of incubation at 42°C for *E. faecalis* and 37°C for the other bacteria, the zone diameters formed around the disks were measured and expressed as millimeter (mm) (Özkan, Demirci, et al., 2021).

### 2.9 Statistical analyses

Two‐sample *t*‐test (TSTT) was used to evaluate the significant differences in the mean values of total phenolic, antioxidant capacity, phenolic compounds, sugars, and organic acids between water and methanol extracts of *J. drupacea* fruit. All statistical analyzes were performed by using MINITAB Software version 14 (Minitab Inc., PA, USA), and results were considered significant at *p* < .05.

## 3 RESULTS AND DISCUSSION

### 3.1 Macro‐ and micromineral composition

The values of phosphorus (P), potassium (K), calcium (Ca), magnesium (Mg), sulfur (S), sodium (Na) macrominerals, and iron (Fe), copper (Cu), mangan (Mn), zinc (Zn), boron (B) microminerals in the fruit fleshy parts of *J. drupacea* are presented in Table 1. It was detected that there were six macro‐ (P, K, Ca, Mg, S, and Na) and five microminerals (Fe, Cu, Zn, Mn, and B) in the fleshy parts of the fruit of *J. drupacea*. Among the macrominerals, the highest concentration was K (15,266 mg/kg), followed by P (776.7 mg/kg), Ca (626.9 mg/kg), Mg (485.0 mg/kg), S (410.8 mg/kg), and Na (34.24 mg/kg), respectively. It was determined that the highest concentration in the micromineral was Fe (28.68 mg/kg), and the lowest concentration was Mn (4.96 mg/kg).

**TABLE 1:** Macro‐ and micromineral elements of *J. drupacea* fruit.

Macroelements (mg/kg DM)		Microelements (mg/kg DM)	
Phosphorus (P)	776.7 ± 0.3	Iron (Fe)	28.68 ± 1.51
Potassium (K)	15,266 ± 147	Copper (Cu)	5.36 ± 0.23
Calcium (Ca)	626.9 ± 6.9	Mangan (Mn)	4.96 ± 0.17
Magnesium (Mg)	485.0 ± 7.3	Zinc (Zn)	7.44 ± 0.25
Sulfur (S)	410.8 ± 8.8	Boron (B)	18.13 ± 0.21
Sodium (Na)	34.24 ± 0.74		

Potassium is one of the most abundant macromineral elements in the fruits of Juniper species and their products such as molasses. It is stated that the most abundant macromineral elements after potassium are P, Ca, and Mg (Akbulut et al., 2008; Akinci et al., 2004; Odabaş‐Serin & Bakir, 2019). Potassium is an important nutrient and constitutes approximately 70% of the positive ions in cells and is essential in regulating the acid–base and water balance in the cells (Senhaji et al., 2022). Ca, K, and Mg are macromineral elements necessary for the repair of worn cells, strengthening of bones and teeth, mechanisms of red blood cells, and body. Since Fe has an important role in oxygen and electron transfer, it is a highly important micromineral element for the human body (Özcan & Akbulut, 2008). Cu and Zn are essential microminerals for human nutrition as they perform a wide variety of functions such as components of enzymatic and redox systems (McLaughlin et al., 1999). Dietary Boron may be an essential micronutrient for animals and humans as it affects the activity of most metabolic enzymes, as well as the metabolism of steroid hormones and various nutrients including Ca, Mg, and vitamin D. It is suggested that boron may also play a role in improving brain functions (Devirian & Volpe, 2003).

The results obtained in our study show that the macro‐ and micromineral elements in *J. drupacea* fruit are of vital importance for human metabolism and therefore human health.

### 3.2 Organic acids and sugar profile

The organic acid results of methanol and water extracts of *J. drupacea* fruit are given in Table 2. Seven organic acids, which were citric acid, tartaric acid, malic acid, lactic acid, formic acid, and propionic acid, were detected in both water and methanol extracts. The highest organic acid was malic acid (1.521%) and the lowest organic acid was citric acid (0.172%) in methanol extract. In water extract of *J. drupacea* fruit, the highest organic acid was malic acid (3.093%), and the lowest organic acid was tartaric acid (0.307%). It was understood that malic acid was the most abundant organic acid in both water and methanol extracts of *J. drupacea* fruit. Therefore, according to this result, it can be said that the dominant organic acid of *J. drupacea* fruit is malic acid.

**TABLE 2:** Organic acid profile of *J. drupacea* fruit methanol and water extracts.

Organic acids (%)	Extracts		*p‐*value
	Methanol	Water	
Citric acid	0.172 ± 0.017	0.702 ± 0.001	.016*
Tartaric acid	0.344 ± 0.045	0.307 ± 0.011	.459
Malic acid	1.521 ± 0.079	3.093 ± 0.132	.044*
Lactic acid	0.799 ± 0.061	0.937 ± 0.070	.283
Formic acid	0.629 ± 0.007	1.111 ± 0.119	.110
Propionic acid	0.384 ± 0.048	0.455 ± 0.037	.345

* Significant level: *p* < .05.

The results of sugar profile in water and methanol extracts of *J. drupacea* fruit were shown in Table 3. Looking at Table 3, it was seen that the most abundant sugar in *J. drupacea* fruit methanol extract was glucose (30.593%), followed by fructose (29.250%) and sucrose (19.673%), respectively. In contrast to the methanol extract, it appeared that the highest sugar concentration in its water extract was fructose, followed by glucose (32.456%) and sucrose (17.545%). This case shows that glucose dissolves better in methanol and fructose dissolves better in water.

**TABLE 3:** Sugar profile of *J. drupacea* fruit methanol and water extracts.

Sugars (%)	Extracts		*p‐*value
	Methanol	Water	
Sucrose	19.673 ± 0.967	17.545 ± 0.791	.251
Glucose	30.593 ± 0.897	32.456 ± 1.131	.319
Fructose	29.250 ± 0.891	41.695 ± 0.652	.040*
Total	79.516 ± 2.760	91.696 ± 0.992	.107

* Significant level: *p* < .05.

So far, no study has been encountered that have determined the organic acid and sugar distribution of *J. drupacea* berries cone extracts. However, there are studies that determine the sugar distribution in some Juniper species such as *J. communis*. However, the sugar profile has been studied in some Juniper species other than *J. drupace*. Falasca et al. (2014) stated that, depending on seasonal changes, the levels of alpha‐glucose, beta‐glucose, sucrose, and fructose in extracts of *J. communis* berries were range of 1.29–52.37, 0.38–26.62, 1.10–15.47, and 0–54.82 mg /g, respectively. It has been reported that the concentration of all sugars except sucrose in *J. communis* berries increases with the winter season.

Fructose is a monosaccharide with a higher sweetness than glucose. Therefore, fructose‐weighted sugar profile gives information about the sweetness value of the product (Carocho et al., 2017; Mao et al., 2019). If it is desired to obtain an extract with high sweetness, it would be more appropriate to extract it with water.

Citric, malic, and tartaric acids are organic acids commonly found in fruits. Citric acid is the dominant organic acid of citrus fruits such as lemon, orange, tangerine, and grapefruit; malic acid is one of the dominant organic acids of fruits such as sour cherry, cherry, and apple, and tartaric acid is of fruits such as grapes. Odabaş‐Serin and Bakir (2019) stated that the titration acidity of *J. drupacea* is in the range of 0.38–0.52%. Titration acidity is a quality parameter that gives information about the total organic acid levels in fruits. The organic acid and sugar balance in fruits are important in terms of not only giving information about their maturity, but also perceiving the flavor of the fruits (Kader, 2008). In addition, sugars and organic acids indirectly contribute to phenolic metabolism by changing the pH and being used as building blocks for phenolic compounds (Perkins‐Veazie & Collins, 2001).

Considering the findings obtained in our study (Table 2), in addition to citric, tartaric, and malic acids, a considerable amount of lactic, formic, and propionic acids was detected in both extracts of *J. drupacea* fruit. These acids are organic acids that are rarely found in fruits and are formed by the fermentation of carbohydrates (Bangar et al., 2022; Ma et al., 2019). The presence of these acids in *J. drupacea* fruit extracts suggests that fermentation may have occurred in the fruit, possibly due to the long waiting period of the fruit after falling under the trees.

### 3.3 Phenolic compounds

#### 3.3.1 Total phenolic (TP) contents

**FIGURE 1:**
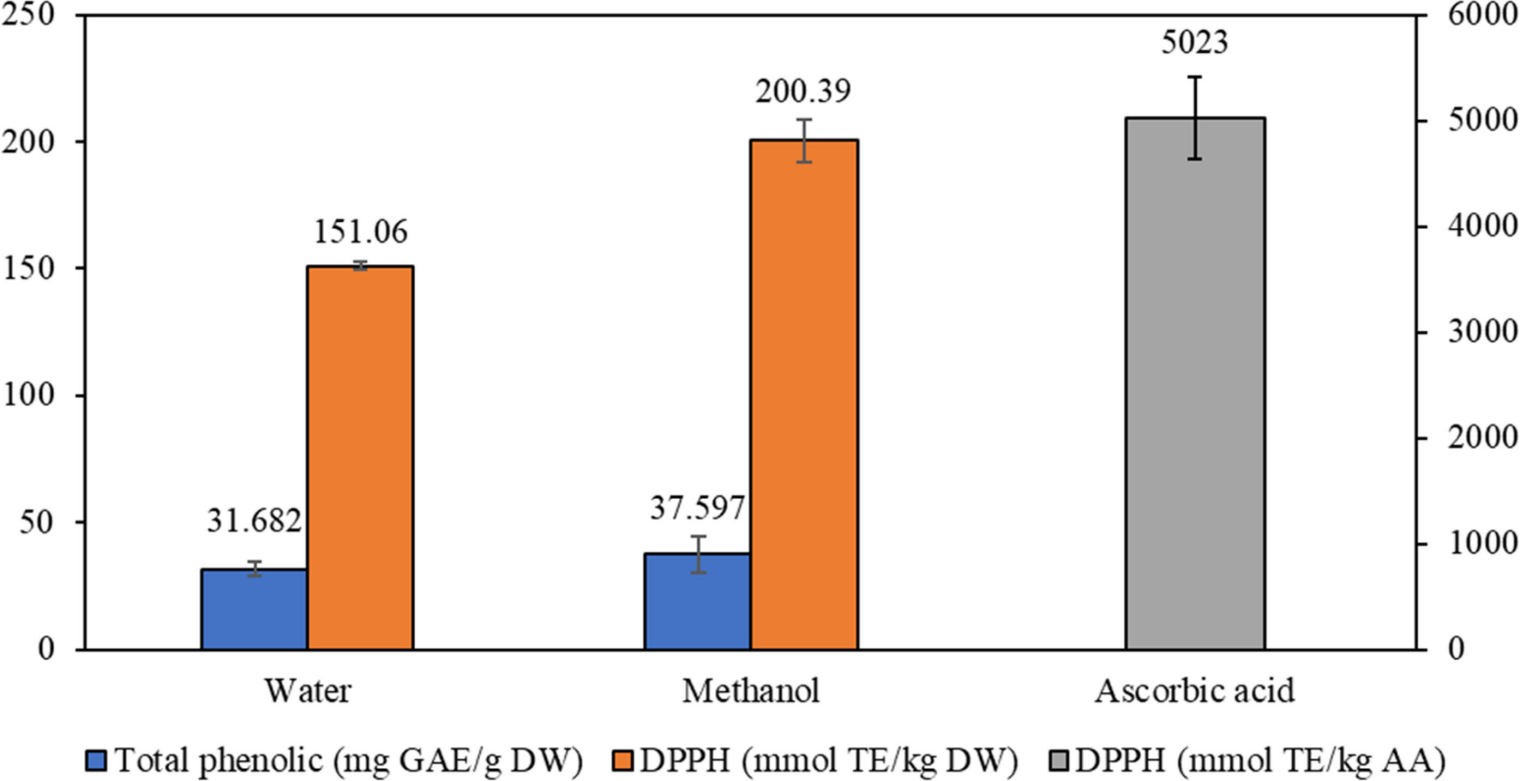
Total phenolic content and DPPH free radical scavenging capacity of the methanol and water extracts of *J. drupacea* fruit.

Data on total phenolic in water and methanol extracts of *J. drupacea* fruit are presented in Figure 1. The amount of total phenolic compounds in the methanol extract (37.597 mg GAE/g DW) was higher than that in the water extract (31.682 mg GAE/g DW). It is understood that methanol may be a better solvent than water in terms of transfer of total phenolic substances.

Akbulut et al. (2008) determined to be 96.5 g/kg the amount of total phenolic substance in molasses obtained from *J. drupacea* fruit. Özkan, Karadağ, et al. (2021) reported that total phenolic content in *J. drupacea* Labill. fruit and molasses were 287.28 ± 17.28 and 372.11 ± 45.72 μg/g, respectively. Considering the total phenolic content obtained in our study, it is seen that it is more than that of Özkan, Karadağ, et al. (2021). Deliorman Orhan et al. (2019) determined that the total phenolic content in methanol extracts of *J. drupacea* fruits was higher than that in the water extract, but lower than that in the ethyl acetate extract. In our study, when we compared water and methanol extracts in terms of phenolic content, it is seen that there is a change similar to the results of Deliorman Orhan et al. (2019).

#### 3.3.2 Individual phenolic compounds

**FIGURE 2:**
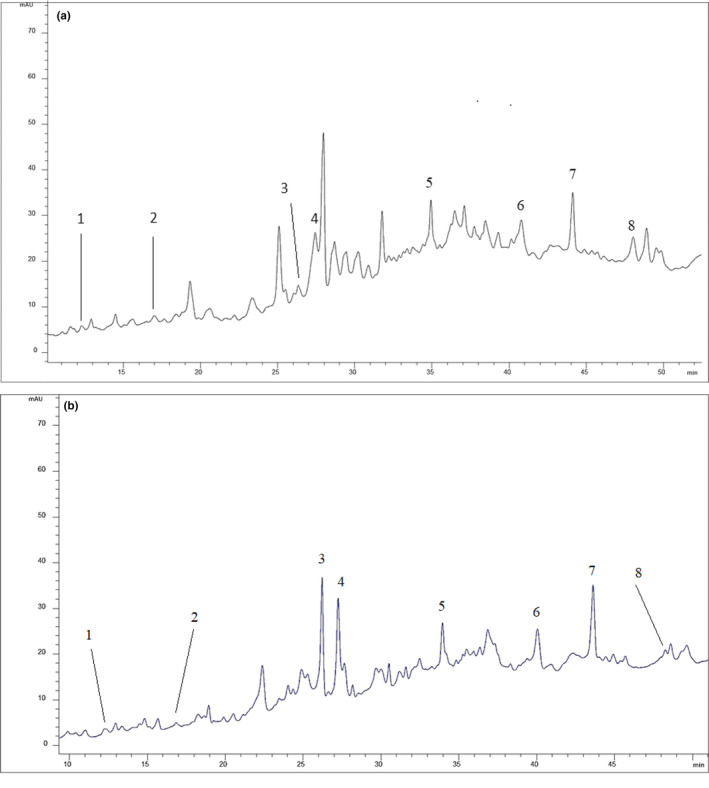
Phenolic profile chromatograms of *J. drupacea* fruit methanol (a) and water (b) extracts. 1: gallic acid; 2: protocatechuic acid; 3: *p*‐hydroxybenzoic acid; 4: catechin; 5: epicatechin; 6: *p*‐coumaric acid; 7: epicatechin gallate; 8: procyanidin‐A2.

The individual phenolic compound distribution and contents of water and methanol extracts of *J. drupacea* fruit are shown in Table 4. Methanol and water solvent extracts of *J. drupacea* fruit present a typical characteristic composition consisting of eight phenolic compounds (Figure 2). Four of these phenolics detected in *J. drupacea* fruit extracts were phenolic acids (gallic acid, protocatechuic acid, *p*‐hydroxybenzoic acid, and *p*‐coumaric acid) and four of them were flavonoids (catechin, epicatechin, epicatechin gallate, and procyanidin A2). Among the phenolic compounds, catechin appears to be the most abundant phenolic compound isolated in all extracts and quantitatively. The highest catechin was determined as 314.88 μg/g in the water extract, followed by epicatechin (176.52 μg/g), epicatechin gallate (94.65 μg/g), procyanidin A2 (74.4 μg/g), p‐coumaric acid (21.24 μg/g), p‐hydroxybenzoic acid (13.70 μg/g), protocatechuic acid (12.01 μg/g), and gallic acid (μg/g).

**TABLE 4:** Phenolic compounds profile of *J. drupacea* fruit methanol and water extracts.

Phenolic compounds (μg/g)	Extracts		*p‐*value
	Methanol	Water	
Gallic acid	11.89 ± 0.81	12.00 ± 1.62	.942
Protocatechuic acid	5.25 ± 0.13	12.01 ± 1.57	.104
*p*‐hydroxybenzoic acid	99.50 ± 6.97	13.70 ± 1.59	.037*
Catechin	300.49 ± 21.6	314.88 ± 42.9	.745
Epicatechin	171.28 ± 10.03	176.52 ± 8.30	.670
*p*‐coumaric acid	28.80 ± 4.57	21.24 ± 1.59	.271
Epicatechin gallate	115.96 ± 3.28	94.65 ± 15.00	.300
Procyanidin A2	4.57 ± 0.46	74.4 ± 5.60	.036*

* Significant level: *p* < .05.

Özkan, Karadağ, et al. (2021) detected a total of eight phenolic compounds in *J. drupacea* Labill. fruit and molasses produced from it, and they determined that the most abundant phenolic compound was protocatechuic acid (77.01 μg/g in berries and 251.65 μg/g in molasses). In another study on *J. drupacea* berries, 18 phenolic compounds were determined, and it was reported that the most abundant phenolic compound among them was protocatechuic acid, a phenolic acid (Miceli et al., 2011). Yaglioglu and Eser (2017) detected four different phenolic compounds in the cones of four different Juniperus species, *J. communis*, *J. excelsa*, *J. foetidissima*, and *J. oxycedrus*, and stated that the most dominant phenolic compound was catechin. The findings of our study regarding the catechin being the most abundant phenolic in the Juniperus species are similar to the results of Yaglioglu and Eser (2017).

The findings of our study showed that the sum of catechin, epicatechin, and epicatechin gallate, which are among the flavanols in the flavonoids group, constituted 79.7% of all determined individual phenolics in water extracts and 81.5% in methanol extracts. As it can be understood from here, flavanols constitute a large part of the phenolics in *J. drupacea* fruits. Catechin, epicatechin, and epicatechin gallate have different benefits for human health. Catechins act as a very important protective natural chemical against cardiovascular and degenerative disorders (Adriouch et al., 2018; Ostojic, 2020). Epicatechin regulates glucose homeostasis and insulin resistance (Cremonini et al., 2019). Epicatechin gallate, which is the conjugate of flavanols with gallic acid (Durazzo et al., 2019), has been studied by many researchers in terms of its potential benefits against many diseases such as cancer and obesity (Bode & Dong, 2009; Casanova et al., 2019; Ostojic, 2020; Wang et al., 2014).

In our study, a large amount of procyanidin A2 was found in water extracts of *J. drupacea* fruit. Procyanidin A2 is a type A proanthocyanin and is oligomers of flavanols. It has been reported that procyanidins might be used in the treatment of obesity due to their positive properties such as regulating lipid absorption, adipose function, and energy expenditure (Salvadó et al., 2015). In addition, proanthocyanidins give positive results in reducing the risk of cancer in animal and in vitro studies, especially colorectal cancer (Koerner et al., 2009; Ouédraogo et al., 2011; Rossi et al., 2010).

### 3.4 Antioxidant capacity

Antioxidant capacity values give information about phytochemicals with antioxidant properties in fruit and vegetables. Alkaloids, ascorbic acid, tocopherols, carotenoids, and phenolic compounds (especially flavonoids) are bioactive phytochemicals with antioxidant capacity. Flavonoids found in fruits and vegetables are bioactive compounds with strong antioxidant capacity due to their free radical scavenging properties (Chigayo et al., 2016; Rezaie et al., 2015).

Some results of studies show that extracts of different Juniperus species have remarkable free radical scavenging capacity (Taviano et al., 2011). In addition, there may be differences in antioxidant capacity in extracts of different solvents. Studies show that methanol extracts have higher free radical scavenging capacity than water extracts (Çoklar & Akbulut, 2016; Coklar & Akbulut, 2017; Taviano et al., 2011).

Figure 1 shows the DPPH radical scavenging capacity results of water and methanol extracts of *J. drupacea* fruit. It was determined that DPPH antioxidant capacity of *J. drupacea* fruit methanol extract (151.06 ± 7.17 mmol TE/kg DW) was higher than that of water extract (200.39 ± 8.47 mmol TE/kg DW). The antioxidant capacity of the standard ascorbic acid, determined by the same method, was 5023 mmol TE/kg AA. The DPPH antioxidant capacity of both extracts was lower than that of ascorbic acid (Figure 1).

Taviano et al. (2011) reported that among the Juniperus species such as *J. communis* L *communis* (*Jcc*), *J. communis* L. var. *saxatilis* Pall. (*Jcs*), *J. drupacea* Labill. (*Jd*), *J. oxycedrus* L. subsp. *oxycedrus* (*Joo*), and *J. oxycedrus* L. subsp. *macrocarpa* (Sibth. & Sm.) Ball. (*Jom*), the highest DPPH antioxidant capacity was in the methanol extract of *Joo*, followed by *Jom* > *Jcs* > *Jd* > BHT > *Jcc*. In the same study, for the water extracts, the order of DPPH scavenging power was *Jom* > *Jcs* > BHT > *Joo* > *Jcc* > *Jd*. The DPPH scavenging capacity of water extracts of *J. drupacea* branches appears to be lower than that of BHT, a potent artificial antioxidant, but its methanol extracts are higher than that of BHT.

### 3.5 Antimicrobial activity

#### 3.5.1 Disk diffusion method

In this study, the results evaluated according to the diameter values of the inhibition zones obtained with the methanol extract of *J. drupacea* fruit against the pathogenic microorganisms tested are shown in Table 5. *L. innocua*, *L. monocytogenes*, *S. carnosus*, and *E. faecalis* gram‐positive pathogens showed an inhibition zone between 5 and 10 mm. Therefore, methanol extract of *J. drupacea* fruit exhibited strong inhibition (++) against these pathogenic bacteria. However, it was determined that the fruit extract of *J. drupacea* did not show any inhibition effect against the tested gram‐negative pathogenic bacteria: *E. coli* and *K. pneumoniae*. At the same time, no‐inhibitory effect of *J. drupacea* fruit extracts could be determined against *S. aureus* and *B. cereus*, which are gram‐positive bacteria.

**TABLE 5:** Antimicrobial activity of methanol extract of *J. drupacea* fruit against various food pathogens by disk diffusion and MIC methods.

Food pathogens	Disk diffusion method	MIC (mg/mL)
*Listeria innocua*	++	25
*Listeria monocytogenes*	++	25
*Staphylococcus aureus*	−	0
*Staphylococcus carnosus*	++	3.12
*Bacillus cereus*	−	0
*Enterococcus faecalis*	++	25
*Escherichia coli*	−	0
*Klebsiella pneumoniae*	−	0

*Note*: (++): the inhibition zones between 5 and 10 mm were considered as a strong inhibition. (−): the regions without diameter formation were evaluated as no‐inhibition.

Miceli et al. (2020) observed that Juniperus leaf extracts showed bacteriostatic activity against *S. aureus* but did not show any activity against gram‐negative bacteria and yeasts they tested and *J. drupacea* gave the most effective result among methanol extracts despite its low phenolic content. Differences in the amount and distribution of phenolic compounds among plant species may also be effective on antimicrobial activity. It has been suggested that although some plant extracts have less phenolic substance than others, their effective antimicrobial activity may be due to the presence of non‐phenolic phytochemicals such as alkaloids, sulfur‐containing phytochemicals, and terpenoids (Barbieri et al., 2017; Miceli et al., 2020).

#### 3.5.2 Minimum inhibitory concentration (MIC)

The MIC results of *J. drupacea* fruit methanol extract is presented in Table 5. It was determined that *J. drupacea* fruit showed a remarkable effect against some gram (+) bacteria tested on methanol extract. Among the gram‐positive bacteria tested, *S. carnosus* (3.12 mg/mL) was the most susceptible, followed equally by *L. innocua*, *L. monocytogenes*, and *E. faecalis* (25 mg/mL). As evident from the results obtained, it was noticed that the methanol extract of *J. drupacea* did not presented any MIC values against any of the gram‐negative bacteria tested, which were *E. coli* and *K. pneumoniae*. At the same time, a similar case was encountered for gram‐positive bacteria tested, which were *S. aureus* and *B. cereus*.

It is stated that the different MIC values of plant extracts against different microorganisms are caused by different bioactive compounds in their structures. Some bioactive compounds such as phenolics are known to be effective natural antimicrobial chemicals against a wide variety of microorganisms (Cowan, 1999; Miceli et al., 2011). Taylor et al. (2005) and Medina et al. (2006) reported that the good antimicrobial properties of *J. drupacea* berries extract compared with *J. communis* var. *communis* (*Jcc*) and *J. communis* var. *saxatilis* (*Jcs*) berries could be due to gallic acid, tyrosol, and catechin only present in *J. drupacea* fruit extracts, absent in *Jcc* and *Jcs* fruit extracts.

Gram‐positive and gram‐negative bacterial species might have different sensitivities to different phenolic distributions in plants due to their differences in membrane structures and, accordingly, cell walls. To be considered an effective antimicrobial agent, a phenolic compound must function at the lipid‐water interface and therefore have a partially hydrophobic property. The fact that some of the phenolic compounds are partially hydrophobic makes it possible for them to act effectively at the membrane interface of gram‐positive bacteria. This can seriously damage the plasticity of the membrane and thus lead to the destabilization of the cell, generally weakening the integrity of the membrane. In this case, it can cause disruption of critical transport processes along with the bacterial membrane (Miceli et al., 2011; Vattem et al., 2004).

### 3.6 Antibiotic susceptibility

Antibiotic susceptibility profile of pathogens tested in this study is presented in Table 6. Ampicillin (10 μg), vancomycin (30 μg), and penicillin G (10 UI) were used to measure the antibiotic resistance of gram‐positive bacteria tested in this study, and netilmicin (30 μg), ciprofloxacin (5 μg), and gentamicin (10 μg) antibiotics were used to measure the antibiotic susceptibility of gram‐negative bacteria. It was determined that against ampicillin, gram‐positive bacteria *L. innocua*, *L. monocytogenes*, *S. carnosus*, and *E. faecalis* were susceptible (S), *B. cereaus* was resistant (R) and *S. aureus* was intermediate (I). While *L. innocua* and *E. faecalis* were resistant (R) to vancomycin, the other bacteria were susceptible. All gram‐positive pathogen bacteria tested were resistance against penicillin G. The gram‐negative bacteria tested, *E. coli* and *K. pneumoniae*, were resistant against the antibiotics netilmicin, ciprofloxacin, and gentamicin. When compared to the effects of tested antibiotics, extracts of *J. drupacea* fruit appear to have significant antimicrobial activities on *L. innocua*, *L. monocytogenes*, *S. carnosus*, and *E. faecalis*.

**TABLE 6:** Resistance of various pathogen bacteria against some antibiotics (inhibition zone, mm).

Food pathogens	Antibiotics		
	Ampicillin	Vancomycin	Penicillin G
**Gram‐positives**			
*L. innocua*	16 (S)	8 (R)	0 (R)
*L. monocytogenes*	23 (S)	16 (S)	13 (R)
*S. aureus*	23 (I)	14 (S)	13 (R)
*S. carnosus*	30 (S)	18 (S)	27 (R)
*B. cereus*	8 (R)	17 (S)	9 (R)
*E. faecalis*	19 (S)	8 (R)	0 (R)

Gram‐negatives	Netilmicin	Ciprofloxacin	Gentamicin
*E. coli*	23 (S)	32 (S)	22 (S)
*K. pneumoniae*	21 (S)	39 (S)	20 (S)

Abbreviations: I, intermediate; R, resistance; S, sensitivity.

## 4 CONCLUSION

*J. drupacea* fruit appears to be rich in minerals, organic acids, sugars, and phenolic compounds. It is understood that *J. drupacea* fruit has a considerable potential in terms of flavonoids, which are especially in the group of phenolic compounds and have a high activity in terms of antioxidant properties. It is noteworthy that catechin, epicatechin, and epicatechin gallate, which are in the class of flavonoids and are very important for human health, constitute around 79.5%–81.5% of the phenolics in *J. drupacea* fruits. When the phenolic compound distribution and their amounts obtained in our study are examined, it is seen that the phenolic component profiles determined in a few studies on *J. drupacea* fruit, and its products are quite different. It is thought that these significant differences may be due to the difference in the region where *J. drupacea* grows, the harvest time, and the extraction method applied to the fruit. Researchers who will study advanced human health originating from the bioactive components of the fruit are advised to collect the *J. drupacea* fruits used in this study in the regions where they are harvested and at the time of harvest, and to use the Soxlet method to obtain the extract.

Six organic acids were determined in both methanol and water extracts of *J. drupacea* berries, and malic acid was the dominant organic acid in both extracts. Since organic acids dissolve better in water, the amounts of individual organic acids determined in aqueous extracts of *J. drupacea* fruit were found to be higher than in methanol extracts. The malic acid ratio determined in the aqueous extract constituted approximately 47% of the total of all individual acids. When *J. drupacea* extracts were evaluated in terms of sugars, sucrose, glucose, and fructose were detected in both extracts, and their amounts were higher in aqueous extracts. It was determined that fructose was the predominant sugar in the aqueous extract, and it constituted 46% of the total sugar.

In the current study, it was determined that *J. drupacea* fruit extracts have very significant antioxidant capacity. When the antioxidant capacities of water and methanol extracts of *J. drupacea* were compared, it was observed that the methanol extracts were higher. The use of methanol in extraction can be recommended because the antioxidant capacity of the methanol extracts of *J. drupacea* is more effective than water extracts. *J. drupacea* methanol extracts showed strong inhibitory effects on the gram‐positive pathogens tested in this study, *L. innocua*, *L. monocytogenes*, *S. carnosus*, and *E. faecalis*. This effect is quite significant when compared to some tested antibiotics. In this respect, it could be useful to use methanol extracts of *J. drupacea* fruit against some pathogens due to its strong antimicrobial properties. The use of *J. drupacea* fruit as molasses and the exposure of sugar‐rich fruits to heat for a long time during the molasses production process results in high amounts of HMF, an intermediate product of the Maillard reaction, which has extremely negative properties for human health. In this respect, it will be extremely useful to use methanol extracts of *J. drupacea* fruit in complementary medicine in terms of being healthier.

## AUTHOR CONTRIBUTIONS

**Hatice Feyza Akbulut:** Data curation (equal); formal analysis (equal); funding acquisition (equal); investigation (equal); methodology (equal); resources (equal); software (equal); supervision (equal); writing – review and editing (equal). **Mehmet Akbulut:** Data curation (equal); formal analysis (equal); investigation (equal); methodology (equal); project administration (equal); resources (equal); supervision (equal); writing – original draft (equal).

## CONFLICT OF INTEREST STATEMENT

The authors declare no conflict of interest.

## ETHICS STATEMENT

This study does not involve any human or animal testing.

## CONSENT TO PARTICIPATE

Written informed consent was obtained from all study participants.

## Data Availability

The data that support the findings of this study are available on request from the corresponding author.
